# Evaluating the neurofilament light chain-derived gap between biological and chronological age in multiple sclerosis and controls

**DOI:** 10.1007/s00415-026-14157-6

**Published:** 2026-09-27

**Authors:** Valerio Nicolella, Carmela Polito, Vincenzo Criscuolo, Marco Varelli, Stefania Fasano, Mariano Fiorenza, Gennaro Pascarella, Antonio Carotenuto, Maria Petracca, Roberta Lanzillo, Giuseppe Castaldo, Vincenzo Brescia Morra, Francesco Salvatore, Daniela Terracciano, Marcello Moccia

**Affiliations:** 1 Department of Neuroscience, Reproductive Science and Odontostomatology, Federico II University of Naples, Naples, Italy; 2 Department of Precision Medicine in Medical, Surgical and Critical Care, University of Palermo, Palermo, Italy; 3 Department of Translational Medical Sciences, Federico II University of Naples, Naples, Italy; 4 Istituto Diagnostico Varelli, Naples, Italy; 5 Department of Molecular Medicine and Medical Biotechnology, Federico II University of Naples, Naples, Italy; 6 Multiple Sclerosis Unit, Policlinico Federico II University Hospital, Naples, Italy; 7 Department of Human Neuroscience, Sapienza University of Rome, Rome, Italy; 8 CEINGE-Biotecnologie Avanzate “Franco Salvatore”, Naples, Italy

**Keywords:** Multiple sclerosis, neurofilament light chain, ageing

## Abstract

**Objective:**

To compare chronological age and neurofilament light chain (NfL)-derived age in people with multiple sclerosis (pwMS) and controls, and to explore clinical correlates of the NfL age gap**.**

**Methods:**

We conducted a real-world, retrospective analysis including 792 pwMS (without evidence of clinical or MRI activity in the previous year and during follow-up) and 302 controls. NfL-derived age was computed using established age-adjusted reference. We used logistic regression models to compare pwMS and controls in chronological and NfL-derived age groups, and linear regression models to evaluate the associations of chronological age and NfL-derived age with clinical variables (Expanded Disability Status Scale (EDSS), disease duration, disease course, disease modifying treatment (DMT) class, number of previous DMTs) and the associations between the NfL age gap (difference between biological and chronological age) and clinical variables.

**Results:**

PwMS showed a higher likelihood than controls of having a NfL-derived age above their chronological age (OR = 1.44;95%CI 1.10—1.87; *p* = 0.007). EDSS, disease duration, and progressive disease course were associated with both chronological and NfL-derived age (all *p* < 0.001). The NfL age gap showed independent associations with EDSS (Coeff = 0.147; 95%CI 0.097–0.197; *p *< 0.001), progressive disease course (Coeff = 0.34; 95%CI 0.16–0.53; *p* < 0.001), and number of previous DMTs (Coeff = 0.06; 95% CI 0.01–0.11; *p* = 0.028), but not with disease duration or DMT class. Similar results were observed in a sub-analysis excluding pwMS with EDSS progression.

**Conclusions:**

MS is associated with a shift in age-associated NfL trajectory, with an older biological profile especially in pwMS with greater disability and progressive course.

**Supplementary Information:**

The online version contains supplementary material available at https://doi.org/10.1007/s00415-026-14157-6.

## Introduction

Identifying strategies to support healthy ageing is crucial for people living with multiple sclerosis (pwMS), as the biological processes of ageing can interact with and potentiate MS-related mechanisms^1^. For this reason, clarifying how ageing trajectories interact with MS pathophysiology is one of the keys to improve care for pwMS ^2^. While chronological age is the most intuitive and easy-to-access measure when examining the relationship between ageing and MS, the concept of biological age may provide a more precise reflection of the pathological changes associated with ageing, offering a clearer window into underlying biological interactions^2–4^. Biological age incorporates the combined impact of genetic, epigenetic, and environmental factors, and implies that individuals of the same chronological age can differ substantially in their biological ageing profiles ^3,5,6^. In MS, biological ageing has been investigated using a variety of approaches, including molecular, cellular, imaging, and clinical metrics.^7–10^

Neurofilament light chain (NfL) is a sensitive indicator of neuroaxonal injury, and its circulating levels increase as a consequence of both age-associated changes and MS-related neuro-axonal loss ^11^. As such, NfL needs to be interpreted within the context of chronological age, but as well provides a biologically grounded measure that can be leveraged to estimate brain biological age and to capture deviations from expected ageing trajectories in people with MS^12^. In this context, age-associated cut-offs that distinguish individuals whose biological age meaningfully exceeds their chronological age may identify those experiencing accelerated ageing^13^. Hereby, we aim to 1) determine whether individuals with stable MS (defined as no evidence of disease activity in the previous year and absence of clinical relapses and/or radiological activity during follow-up) show evidence of accelerated ageing, operationalized through the gap between NfL-derived age and chronological age; and 2) investigate how demographic and clinical characteristics relate to chronological age, NfL-derived age, and the NfL age gap, thereby identifying factors associated with accelerated NfL-derived age in MS, with specific regard to EDSS progression during follow-up.

## Methods

### Study design and population

This retrospective study was conducted at the MS Clinical Unit of the Federico II Policlinico University Hospital, Naples, Italy*.* The study was approved by the Campania-3 Ethics Committee (158/25).

We included consecutive pwMS aged >18 years with no evidence of disease activity (NEDA3) in the previous year (defined by the absence of clinical relapses, MRI activity, and EDSS progression), from September 2023 to July 2025, regardless of Expanded Disability Status Scale (EDSS) or treatment status, as assessed through routine clinical follow-up visits and radiological evaluations. The first available pNfL measurement was considered for the study (no longitudinal NfL measurements were included in the present analyses). Following study extraction, patients underwent routine clinical follow-up approximately every 6 months (at least until Jul 2026); follow-up visits were subsequently used to assess disease activity and EDSS progression.

Individuals with a history of other neurological disorders or head trauma were excluded, as well as patients who subsequently experienced clinical relapses and/or radiological activity, whereas EDSS progression during follow-up was allowed but recorded for further sub-analyses^14–16^.

Patients were asked to participate to the study at their scheduled neurological consultation and blood drawn. Clinical relapses were defined as new, worsening, or recurrent neurological symptoms lasting at least 24 h in the absence of infection, fever, or adverse reaction to prescribed medication^17^. MRI activity was defined as at least one new or enlarging T2 lesions and/or T1 gadolinium-enhancing lesions, as documented in the official radiology report and confirmed by a neuroradiologist^17^. EDSS progression was defined as an increase of 1.5 points if baseline EDSS was 0, 1 point if baseline EDSS was between 1.0 and 5.5, or 0.5 points if baseline EDSS was > 5.5 ^16^. We also recruited consecutive controls, attending their scheduled blood drawn for preventative purposes, in the absence of history of neurological diseases or head trauma^14^.

All participants signed informed consent authorizing the use of anonymized data in line with data protection regulation (GDPR EU 2016/679). The present study was performed in accordance with good clinical practice and the Declaration of Helsinki.

### Demographic and clinical variables

For both MS cases and controls, we collected age and sex. We then classified MS cases and controls in chronological age groups based on chronological age ranges 18 to 50, 51 to 60, 61 to 70 years, and > 70 years, as suggested Simrén and colleagues^13^.

For MS cases we collected the following variables: expanded disability status scale (EDSS), MS disease duration (time from reported onset to assessment), descriptor of disease progression (relapsing, progressive), current disease modifying treatment (DMT), duration of current DMT and number of previous DMTs. Current DMT was defined according to treatment exposure at the baseline visit, corresponding to the time of blood sampling. Subsequent DMT changes during follow-up were not considered in the present analyses. For statistical purpose, DMTs were categorised into monoclonal antibodies (alemtuzumab, natalizumab, ocrelizumab, and ofatumumab), oral therapies (cladribine, dimethyl fumarate, fingolimod, siponimod, ozanimod, ponesimod, and teriflunomide), and injectables (peg-interferon beta, interferon beta, and glatiramer acetate)^18^. During the follow-up, we further collected relapses, MRI activity and EDSS progression.

### Neurofilament light chain measurements

Fasting blood samples were obtained on the same day as the clinical assessments. Plasma samples were collected simultaneously in BD Vacutainer™ tubes containing ethylene-diamine-tetra-acetic acid (EDTA) as anticoagulant. Blood samples were centrifuged within 3 h after draw at 1100 rpm for 10 min, aliquoted into polypropylene tubes, and stored at –80 °C. pNfL quantification (pg/mL) was performed according to the manufacturers’ instructions using the Fujirebio Lumipulse® G NfL Blood assay. Results were analyzed in singlicate and expressed in picograms per milliliter (pg/mL) according to standard laboratory practice.

We converted Lumipulse-measured NfL values to their SIMOA equivalents using the published conversion algorithm; subsequently, SIMOA-equivalent concentrations were transformed into the equivalents of the assay kits used by Simren and colleagues^13,19,20^. Finally, both pwMS and controls were classified according to age-specific pNfL cut-offs recommended by Simrén et al. into predefined age bands (5–17, 18–50, 51–60, 61–70, and > 70 years).

In addition, an “over” category was defined for individuals whose pNfL levels exceeded the age-specific reference cut-off within their chronological age group; this classification was defined as NfL-derived age.^13^

As the comparison was performed between predefined chronological age categories and NfL-derived age categories, categories were converted into ordinal numerical ranks reflecting their position along the age continuum. The NfL age gap was calculated as the difference between the ordinal rank assigned to the NfL-derived age category and the ordinal rank assigned to the chronological age category. Therefore, positive values indicate assignment to an older NfL-derived age category than expected, whereas negative values indicate assignment to a younger category. The resulting NfL age gap ranged from −3 to + 4*,* reflecting the possible category shifts between these two measures.

### Statistical analyses

Mean (and standard deviation) were calculated for chronological age, pNfL concentrations, disease duration and follow up duration. Median (and range) were calculated for EDSS and number of follow-up visits. Number (and percent) were calculated for sex, age categories, NfL-derived age categories, age gap, descriptor of disease progression, EDSS worsening and disease-modifying therapy (DMT) groups. Group differences between pwMS and controls were assessed using independent-samples t-tests for continuous variables and chi-square tests for categorical variables.

To determine whether pwMS are biologically older than expected compared with controls, we used ordered logistic regression models with NfL-derived age as the dependent variable and chronological age group as the independent variable. The proportional-odds assumption was formally tested using Wald tests for each model. When this assumption was not violated, ordered logistic regression was retained. When the proportional-odds assumption was violated, generalized ordered logistic models were applied using partial proportional-odds specifications to relax the constraint where necessary. Models were estimated separately in the pwMS subgroup and in the control group to compare the strength of the relationship between chronological age group and NfL-derived age in each group. Then, we also tested the interaction between disease status (pwMS vs controls) and chronological age group to evaluate whether case–control differences in *NfL-derived age* classification vary across age groups.

To evaluate whether in pwMS NfL-derived age is more strongly associated with clinical variables than chronological age, we performed ordered logistic regression models including, in turn, chronological age group and NfL-derived age group as dependent variable; and, in turn, clinical variables, including EDSS, disease duration, descriptor of disease progression (relapsing, progressive), DMT class and number of previous DMTs as independent variables; the proportional-odds assumption was tested for each model, and generalized ordered logistic models were used whenever the assumption was violated. Then, to further characterize the biological relevance of the NfL age gap, linear regression models were used including NfL age gap as dependent variable and, in turn, clinical variables, including EDSS, disease duration, descriptor of disease progression (relapsing, progressive), DMT class and number of previous DMTs as independent variable, with age and sex included as covariates. Although the NfL age gap is a bounded discrete variable derived from ordinal age categories (range −3 to + 4), it was treated as an approximately continuous outcome. In addition, multivariable models including these clinical variables simultaneously were fitted to assess their independent associations in both the ordered logistic and linear regression analyses, applying generalized ordered logistic models when the proportional-odds assumption was not met.

Cox proportional hazards models were used to assess the association between NfL age gap and risk of EDSS progression, adjusting for age and sex. Then, a sensitivity analysis was performed in patients without confirmed EDSS progression, using the same statistical models and covariates as in the main analysis^15^.

Results were reported as coefficients (Coeff), odds ratio (OR), hazard rates (HR), 95% confidence intervals (95% CI), and p-values, as appropriate. Distribution of variables and residuals was checked using both graphical and statistical methods. Statistical analyses were performed using Stata 15.0 (StataCorp, College Station, TX, USA). Results were considered statistically significant if *p* < 0.05. Data visualizations and figures were generated using Stata 15.0 (StataCorp, College Station, TX, USA) and the internal UNINA Copilot AI system under the supervision of the authors.

## Results

### Study population

Among 889 pwMS screened for eligibility, 792 were included in the final analyses. Ninety-seven patients were excluded, including 46 who developed clinical relapses and/or radiological activity during follow-up and 51 who met other exclusion criteria. Together with 302 controls, the final study population comprised 1094 participants. All included pwMS were clinically and radiologically stable, with NEDA3 status during the previous year and no subsequent clinical relapses or radiological activity during follow-up.

**Fig. 1:**
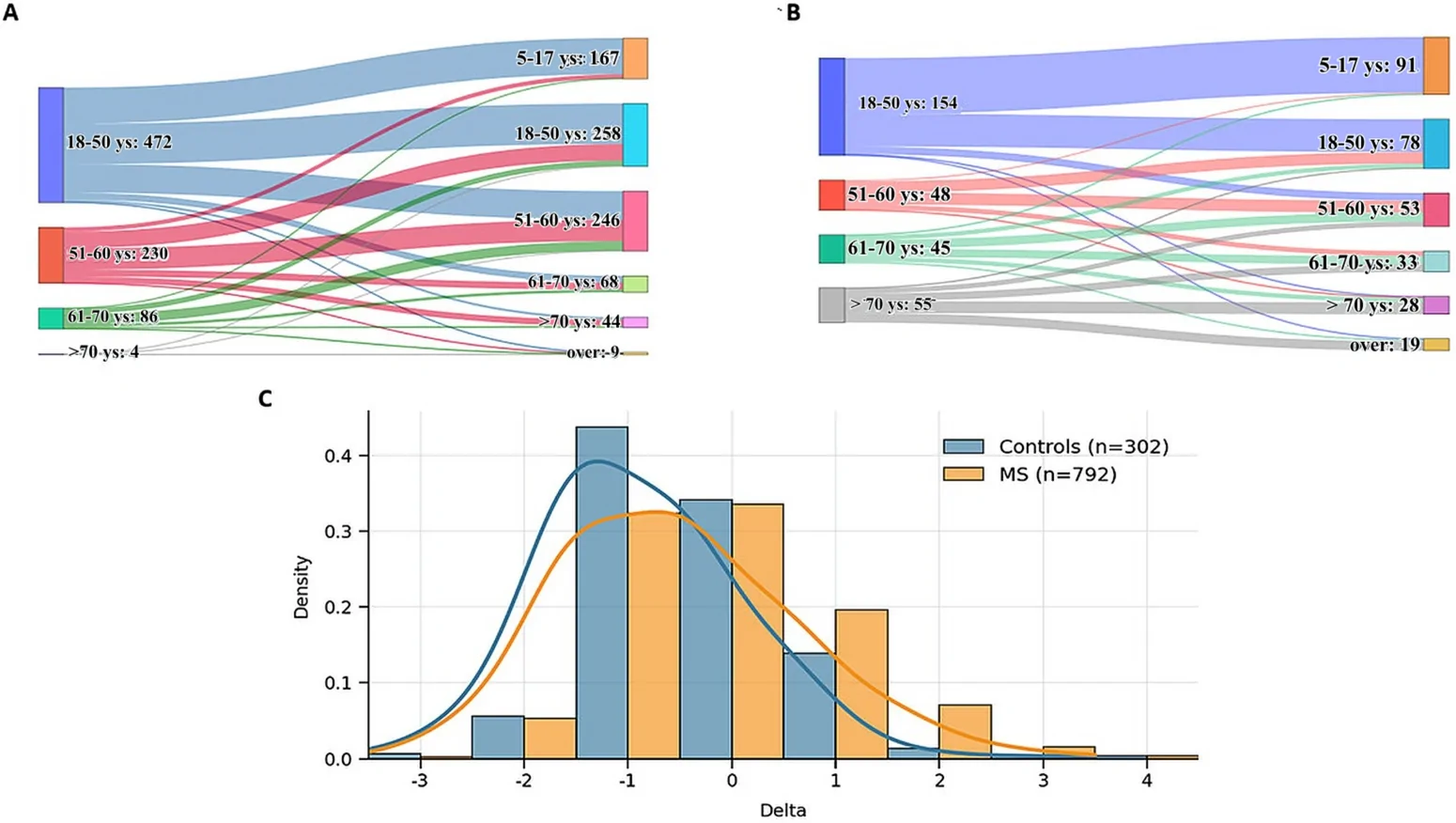
Differences between chronological age and NfL-derived age. Sankey diagrams show the redistribution of participants from chronological age groups to plasma NfL-derived age categories in pwMS (**A**) and controls **B**. Chronological age groups and NfL-derived age categories were defined according to age-specific pNfL cut-offs (5– < 17, 18– < 50, 51– < 60, 61– < 70, and ≥ 70 years). The “over” category indicates individuals whose NfL-derived age category exceeds their corresponding chronological age group based on these cut-offs. Thus, participants classified as “over” have pNfL levels above the expected threshold for their age band, reflecting a higher NfL-derived age relative to their chronological age. Panel C shows the distribution of age gaps across pwMS (orange) and controls (blue), displayed through overlaid histograms and kernel density estimates

Females represented the majority in both groups (MS: 503; controls: 157). Controls were older than MS participants (*p* < 0.001), with a mean age of 50.6 ± 18.1 years, compared with 47.4 ± 11.1 years in the MS group, and with subsequently higher pNfL values (*p* < 0.001). Demographic, clinical, and treatment variables are reported in Table 1. Group differences between pwMS and controls were explored by comparing the distribution of chronological age categories, NfL-derived age categories, and the age gap (defined as the difference between NfL-derived age and chronological age), and are reported in Table S1. Figure 1 illustrates the redistribution of participants from chronological age groups to NfL-derived age categories, together with the distribution of age gaps across cohorts. Supplementary materials (Tables S2**–S3** and Figure S1) present the same results restricted to pwMS who did not show EDSS progression during follow-up.

**Table 1:** Demographic, laboratory, and clinical features of MS cases and controls. Statistically significant differences between groups (p<0.05)

Variable	MS (*n* = 792)	Controls (*n* = 302)	*p* value	Total (*n* = 1094)
Age, years (mean ± SD)	47.43 ± 11.07	50.61 ± 18.11	**<0.001**	48.31 ± 13.46
Sex, female (n, %)	503 (63.5%)	157 (52.0%)	**<0.001**	660 (60.3%)
Disease duration, years (mean ± SD)	16.62 ± 9.72	—	—	—
Follow-up from blood draw, years (mean ± SD)	1.98 ± 0.48	—	—	—
Follow up visits, median (range)	4 (1–6)			
EDSS, median (range)	3 (1–8)	—	—	—
Disease course				
Relapsing (n, %)	588 (74.2%)	—	—	—
Progressive (n, %)	204 (25.8%)	—	—	—
EDSS progression during follow up (n, %)	100 (12.6%)	—	—	—
DMT group				
Injectable DMTs (n, %)	63 (7.95%)	—	—	—
Oral DMTs (n, %)	485 (61.24%)	—	—	—
Monoclonal antibodies (n, %)	244 (30.81%)	—	—	—
PNfL (pg/ml, mean ± SD)	12.01 ± 8.45	14.71± 15.33	**<0.001**	12.76 ± 10.85
Simoa-converted NfL (pg/ml, mean ± SD)	12.76± 10.85	12.59± 7.94	**<0.001**	13.29 ± 10.20

Statistically significant differences between groups (*p* 0.05) are highlighted in bold

### MS cases vs Controls

The proportional-odds assumption was not violated in either controls (*p* = 0.53) or pwMS (*p* = 0.056), nor in the comparison between MS and controls (*p* = 0.36). Therefore, ordered logistic regression was used for all group-comparison analyses.

There was a significant association between NfL-derived age and chronological age group in both pwMS (OR = 2.67; 95%CI 2.23–3.20; *p* < 0.001) and controls (OR = 7.23; 95%CI 5.40–9.69; *p* < 0.001), though the association was stronger in the latter group. When directly comparing MS and controls, the probability of having a NfL-derived age exceeding chronological age group was 45% higher in individuals with MS than in controls (OR = 1.44; 95%CI 1.10—1.87; *p* = 0.007).

Results were consistent in sensitivity analyses restricted to pwMS without EDSS progression during follow-up. The proportional-odds assumption was met in controls but was marginally violated in pwMS (*p* = 0.049). Detailed results of the ordinal logistic regression analyses are presented in Table S4.

### Clinical variables

When testing the proportional-odds assumption for clinical variables, the constraint was violated for disease duration (*p* < 0.01). Therefore, models including disease duration were fitted using generalized ordered logistic regression with partial proportional-odds constraints.

EDSS was associated with both NfL-derived age (OR = 1.51; 95% CI 1.40–1.63; *p* < 0.001) and chronological age group (OR = 1.57; 95% CI 1.45–1.71; *p* < 0.001). Likewise, each one-point increase in EDSS was associated with a higher NfL age gap (Coeff = 0.147; 95% CI 0.097–0.197; *p* < 0.001). Disease duration was associated with NfL-derived age across all cumulative thresholds of the generalized ordered logistic model (all *p* ≤ 0.001), but not with the NfL age gap. Progressive disease course was associated with both NfL-derived age (OR = 2.56; 95% CI 1.91–3.43; *p* < 0.001) and chronological age group (OR = 2.70; 95% CI 1.99–3.67; *p* < 0.001). Participants with a progressive course also showed a significantly higher NfL age gap (Coeff = 0.34; 95% CI 0.16–0.53; *p* < 0.001). Compared with injectable DMTs, oral DMTs were associated with both lower NfL-derived age (OR = 0.54; 95% CI 0.34–0.86; *p* = 0.009) and lower chronological age group (OR = 0.47; 95% CI 0.28–0.78; *p* = 0.003), whereas monoclonal antibodies were associated only with chronological age group (OR = 0.49; 95% CI 0.28–0.78; *p* = 0.003). DMT duration was associated with both NfL-derived age and chronological age group (both *p* < 0.001), but not with the NfL age gap. Conversely, although the number of previous DMTs was not associated with either age measure, each additional previous DMT was associated with a larger NfL age gap (Coeff = 0.06; 95% CI 0.01–0.11; *p* = 0.028).

**Fig. 2:**
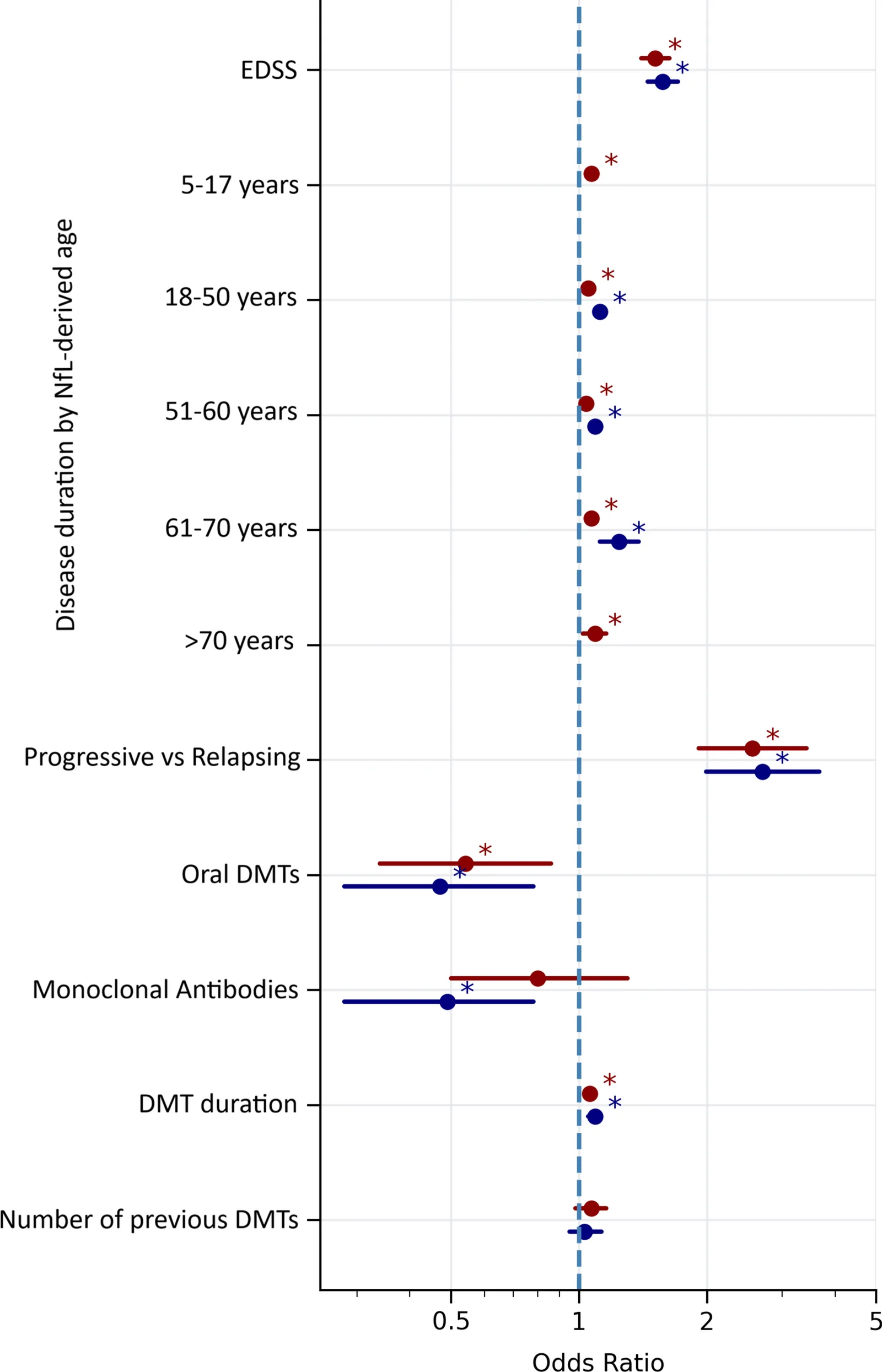
Direct comparison of clinical correlates for chronological age and NfL-derived age. Forest plot shows odds ratios (ORs), 95% confidence intervals (95% CIs), and p values for the associations of clinical variables with chronological age group (blue) and NfL-derived age group (red). For disease duration, estimates for NfL-derived age correspond to the cumulative thresholds of the generalized ordered logistic model. Significant associations (*p* < 0.05) are marked with an asterisk (*)

Detailed estimates are reported in Table 2. Figure 2 shows the associations between clinical variables and both chronological age and NfL-derived age.Similar trends were observed after excluding pwMS with EDSS progression (Table S5).

**Table 2:** Associations between clinical predictors and age metrics. Table shows coefficients (Coeff), odds ratio (OR), 95% confidence intervals (95% CI), and p values for the associations between clinical predictors and chronological age group, NfL-derived age group and brain age gap as dependent variables. and clinical variable (disease duration, EDSS, descriptor of disease progression, DMT group, DMT duration, number of previous DMTs) in turn as independent variables. Covariates for regression models were age and sex. For disease duration, ORs represent threshold-specific cumulative odds ratios derived from generalized ordered logistic models Significant results (*p* < 0.05) are reported in bold

		OR/Coeff	95% CI Lower	95% CI Upper	*p* value
EDSS	Chronological age group	1.57	1.45	1.71	**<0.001**
	NfL-derived age	1.51	1.40	1.63	**<0.001**
	NfL age gap	0.16	0.11	0.20	**<0.001**
Disease Duration (18–50 yrs)	Chronological age group	1.12	1.10	1.14	**<0.001**
	NfL-derived age	1.05	1.03	1.06	**<0.001**
	NfL age gap	0.01	–0.00	0.02	0.164
Disease Duration (51–60 yrs)	Chronological age group	1.09	1.06	1.11	**<0.001**
	NfL-derived age	1.04	1.02	1.06	**<0.001**
	NfL age gap	0.01	–0.00	0.02	0.164
Disease Duration (61–70 yrs)	Chronological age group	1.24	1.12	1.38	**<0.001**
	NfL-derived age	1.07	1.04	1.10	**<0.001**
	NfL age gap	0.01	–0.00	0.02	0.164
Disease Duration (> 70 yrs)	Chronological age group	—	—	—	—
	NfL-derived age	1.09	1.02	1.16	**<0.001**
	NfL age gap	0.01	–0.00	0.02	0.164
Clinical course (Progressive vs Relapsing)	Chronological age group	2.70	1.99	3.67	**<0.001**
	NfL-derived age	2.56	1.91	3.43	**<0.001**
	NfL age gap	0.34	0.16	0.53	**<0.001**
DMT group (ref: Injectable)					
Injectable (reference)					
Oral	Chronological age group	0.47	0.28	0.78	**0.003**
	NfL-derived age	0.54	0.34	0.86	**0.009**
	NfL age gap	–0.07	–0.37	0.22	0.616
Monoclonal	Chronological age group	0.49	0.28	0.78	**0.003**
	NfL-derived age	0.80	0.50	1.30	0.374
	NfL age gap	0.17	–0.14	0.48	0.286
DMT duration	Chronological age group	1.09	1.05	1.12	**<0.001**
	NfL-derived age	1.06	1.03	1.09	**<0.001**
	NfL age gap	0.01	–0.01	0.03	0.308
Number of previous DMTs	Chronological age group	1.03	0.95	1.13	0.452
	NfL-derived age	1.07	0.98	1.16	0.132
	NfL age gap	0.06	0.01	0.11	**0.028**

Statistically significant differences between groups (*p* 0.05) are highlighted in bold

### Multivariable models with clinical variables

In multivariable models including EDSS, disease duration, disease course, age and sex, the proportional odds assumption was not violated overall (*p* = 0.35 for chronological age; *p* = 0.16 for NfL-derived age), except for disease duration (*p* < 0.001 and *p* = 0.038, respectively) which was therefore modeled using partial proportional odds.

EDSS was independently associated with chronological age group (OR = 1.44; 95% CI 1.27–1.64; *p* < 0.001) and NfL-derived age (OR = 1.43; 95% CI 1.28–1.60; *p* < 0.001). Disease duration was associated with both chronological age group and NfL-derived age. For chronological age group, significant associations were observed across the cumulative thresholds of the generalized ordered logistic model, corresponding to the transitions between age categories (5–17 years: OR = 1.12; 95% CI 1.10–1.14; 18–50 years: OR = 1.09; 95% CI 1.06–1.11; 51–60 years: OR = 1.24; 95% CI 1.12–1.38; all *p* < 0.001). Similarly, for NfL-derived age, threshold-specific associations were observed across the cumulative thresholds corresponding to the NfL-derived age categories (5–17 years: OR = 1.06; 95% CI 1.03–1.08; *p* < 0.001; 18–50 years: OR = 1.03; 95% CI 1.01–1.05; *p* < 0.001; 51–60 years: OR = 1.02; 95% CI 1.00–1.04; *p* = 0.036; 61–70 years: OR = 1.05; 95% CI 1.02–1.08; *p* = 0.003; and ≥ 70 years: OR = 1.07; 95% CI 1.00–1.14; *p* = 0.057). These thresholds correspond to the age-specific reference intervals used to define chronological age and NfL-derived age categories and should not be interpreted as the actual ages of study participants.

Disease course was not independently associated with neither chronological age group (OR = 0.80; 95% CI 0.50–1.28; *p* = 0.357) nor NfL-derived age (OR = 0.91; 95% CI 0.61–1.36; *p* = 0.647). In linear regression analyses, EDSS was associated with a higher NfL age gap (Coeff = 0.15; 95% CI 0.08–0.21; *p* < 0.001), whereas disease duration (*p* = 0.557) and disease course (*p* = 0.927) were not.

In univariable Cox regression analysis, the NfL-derived predictor was not associated with risk of progression (HR 1.01, 95% CI 0.99–1.03, *p* = 0.29). In multivariable models adjusted for age and sex, the association remained non-significant (HR 1.00, 95% CI 0.98–1.02, *p* = 0.99).

### Sensitivity analyses

Results were consistent in sensitivity analyses including pwMS without EDSS progression during follow-up. Both chronological age and NfL-derived age remained independently associated with EDSS (OR 1.51 and 1.45, respectively; *p* < 0.001) and with disease duration, with a non-linear pattern for chronological age and a consistent effect for NfL-derived age. Disease course was not independently associated with either age metric (OR 0.60 and 0.87; *p *= 0.063 and *p *= 0.542, respectively). In linear models, EDSS was associated with a higher NfL age gap (Coeff = 0.15, 95% CI 0.08–0.21; *p* < 0.001), whereas disease duration (Coeff = 0.01, 95% CI −0.00 to 0.02; *p *= 0.348) and disease course (Coeff = 0; 95% CI–0.22–0.31; *p* = 0.743) were not.

## Discussion

In our real-world study, we showed that pwMS have older NfL-derived age than controls even though absolute NfL levels were overall higher in the unmatched control group than in pwMS, suggesting that MS-related mechanisms induce accelerated brain ageing as a result of chronic neuro-axonal injury, with related clinical associations. Of note, the association between chronological age and NfL-derived age was present in both controls and pwMS, though substantially weaker in the latter, reflecting a shift toward an older biological profile.

Although NfL is known to increase with age and disease activity in MS, as captured by established z-score approaches based on large healthy reference cohorts, the present “NfL-age” framework provides complementary information^12^. While z-scores quantify deviations from age-adjusted normative values, they do not translate these deviations into an interpretable biological age range and are primarily used as predictors of subsequent disease activity^21^. In contrast, our cohort included patients without recent and subsequent disease activity, allowing us to investigate NfL-related processes independently of acute inflammatory dynamics. Importantly, the plasma NfL cutoffs adopted from Simrén et al. represent high-percentile thresholds (approximately the upper 95th percentile), conceptually similar to z-score-based extremes^13,22^. As such, they are intended to identify “red flag” values rather than to define physiological aging trajectories, further supporting the interpretation of our approach as capturing relative neuroaxonal burden rather than normative aging patterns.

Biological age can be estimated using multimodal approaches, including epigenetic clocks and telomere length, which capture systemic ageing processes. In contrast, our study relied on plasma NfL as a marker of chronic neuroaxonal injury (especially considering that we included pwMS with NEDA3 in the previous year and controls without history of neurological diseases). Thus, NfL is meant to reflect a brain-specific dimension of biological ageing rather than a comprehensive systemic ageing construct. This interpretation is consistent with the findings of Oh and colleagues showing that brain-derived plasma proteins, most prominently NfL, were able to capture a distinct ageing trajectory that was only weakly correlated with organism-wide ageing markers^6^. Moreover, in their work, plasma-based brain age gaps showed only weak correspondence with MRI-derived estimates of brain ageing, suggesting that blood-based neuroaxonal markers capture molecular and cellular aspects of brain ageing that are not fully reflected by structural imaging.^23^. As such, the use of a blood-based neuroaxonal biomarker offers a scalable and clinically accessible approximation of brain ageing in routine practice.

In our study, looking at differences between pwMS and controls, there was correspondence between chronological age and NfL in around 34% of both people with MS and controls, confirming that NfL increases with age ^11^. However, the markedly stronger association observed in controls indicates that the age-related increase in NfL followed a more predictable trajectory in the absence of observable neurological diseases. By contrast, MS-related neuroaxonal injury appears to introduce variability that weakens this relationship, suggesting that NfL reflects not only population-level ageing, which is an association driven phenomenon, but also disease-related mechanisms ^24^–^25^. Altogether, these results indicate that MS disrupts brain ageing patterns, captured by NfL, leading to a NfL age gap from the divergence between NfL-derived and chronological age ^2^. Our study did not include patients with recent or subsequent clinical or radiological disease activity, thereby reducing the likelihood that elevated NfL levels were driven by acute inflammatory processes^26^. In this context, NfL levels are more likely to reflect chronic neurodegenerative mechanisms rather than transient disease activity^27^. Moreover, sensitivity analyses performed in patients without EDSS progression yielded consistent results, confirming the findings observed in the main study population.

So far, the brain age paradigm has been estimated in MS using MRI. In particular, the brain age gap was calculated as the difference between chronological age and MRI-derived estimated brain age, and was found to be higher in MS than controls, and associated with MS clinical features, thus reflecting the overlap between biological brain age- and MS-related changes^4,28–30^. In their MRI-based study, Chai and colleagues demonstrated a markedly increased brain age gap in MS compared with controls and other neurological disorders. Similarly, we observed that individuals with MS were significantly more likely to exhibit an NfL-derived age exceeding their chronological age^28^. Our findings also align with those of Pontillo and colleagues, who showed that neurodegeneration in MS progresses faster than expected based on disease duration alone, as captured by disease-duration gap models^29^. Together, these observations suggest that accelerated ageing in MS is more closely related to disease severity and progression than to disease duration or treatment exposure^4,5,26,31^. This stressed the concept that ageing is made of molecular and cellular alterations even before clinical over situation may appear^4,5^.

As the burden of age-related changes is not fully captured by routine clinical measures, pNfL may provide an accessible tool to estimate age-related neuroaxonal changes. On the current ground, pNfL should be considered a dynamic marker of neuroaxonal injury relative to age rather than a direct surrogate measure of brain biological age^35,36^. Nevertheless, the divergence between chronological age and NfL-derived age, together with its associations with disability and progressive disease course, suggests that it may capture age-related biological processes that overlap with mechanisms implicated in accelerated brain ageing in MS^23,37^.

NfL-derived age confirmed established associations of NfL, thus providing clinical significance. In particular, a higher NfL age gap was associated with higher EDSS scores and a progressive disease course, suggesting accelerated neuroaxonal ageing^9,38^. In contrast, disease duration, DMT exposure, and the number of prior DMTs showed weak or non-significant associations, likely because these variables are closely linked to disease phenotype, with progressive MS generally characterized by longer disease duration and greater treatment exposure^39^. From a mechanistic perspective, NfL is well established as a marker of neuroaxonal injury, disability accumulation, progressive disease course, and neurodegeneration in MS. Accordingly, the observed associations between the proposed ageing metric and clinical severity measures, such as EDSS or progressive course, may in part reflect intrinsic properties of the biomarker itself rather than an entirely independent construct of ageing. However, this does not undermine the validity of our approach. Rather, it highlights that the proposed NfL-derived age captures a biologically meaningful dimension of neuroaxonal burden that is closely intertwined with disease severity. This interpretation is further supported by our findings showing that the association between disease duration and age categories varies across strata, with slightly higher odds ratios observed in older age groups, suggesting a cumulative effect of disease duration that becomes more apparent at higher ages. The inclusion of a clinically and radiologically stable MS cohort minimized the potential influence of acute inflammatory disease activity on NfL levels, allowing a more specific evaluation of age-related neuroaxonal burden in MS. Whether these findings extend to patients with ongoing inflammatory disease activity remains to be established and should be investigated in future studies^40^.

Limitations of our study include that controls were older than pwMS (resulting into higher pNfL); however, we specifically focused on differences between chronological and NfL-derived age, and overall age differences between cases and controls were out of scope. A further limitation is the lack of information on comorbidities and BMI, which may confound the relationship between chronological age, MS-related damage and NfL levels, as well as the association between NfL levels and disability^12,41–44^. Although individuals with a history of neurological disorders or head trauma were excluded, comorbidities, particularly cardiovascular and metabolic disorders, may still influence circulating NfL levels and complicate the interpretation of NfL-derived biological age.

^42,43^. Furthermore, NfL-derived age remains an exploratory metric, as NfL levels may be influenced by disease-specific and other biological factors, and therefore require further validation^14^.

In addition, BMI may act not only as a proxy for metabolic and inflammatory status but also as an indicator of volume of distribution, potentially affecting circulating NfL concentrations independently of neuroaxonal injury^12,44,45^. NfL levels increase significantly with age, following a non-linear trajectory with a steeper rise around the age of 50 in both the general population and individuals with neurological diseases^12^. In this context, we acknowledge that the 18–50 age range adopted in our study is relatively broad, which may limit the precision of age-related analyses, particularly when relying on the broader NfL cut-offs it also introduces variability in the age gap values especially among patients near the upper and lower limits of the intervals ^13^. NfL age categories were derived from previously published age-specific SIMOA-derived reference values rather than cohort-specific normative data. Although these cut-offs were established in large independent cohorts, the distribution of NfL-derived age in our healthy controls did not perfectly overlap with chronological age, particularly within the broad 18–50 years category. This discrepancy may reflect both the wide age span covered by this interval, the use of a conversion formula to derive SIMOA-equivalent NfL values, and population-specific differences in NfL distributions. In addition, several factors beyond age, including comorbidities, lifestyle characteristics, and social determinants of health such as educational attainment, may contribute to inter-individual variability in NfL levels and are not accounted for in the reference model^46^. However, our objective was not to validate the reference ranges themselves or develop a novel biological age clock, but rather to investigate deviations from established age-adjusted NfL categories in people with MS. Also, we found no association between NfL age gap and the risk of EDSS progression. This observation is consistent with growing evidence suggesting that, compared with NfL, disability accumulation may be more closely related to biomarkers reflecting astroglial activation and chronic neurodegenerative processes, such as GFAP^49–51^. Given the larger NfL age gap observed in progressive MS, the inclusion of GFAP could have provided complementary information regarding progression-related pathology and potentially improved our ability to capture biological mechanisms underlying disability worsening. Future studies integrating both NfL and GFAP may help refine biological ageing models and improve prediction of disability progression ^6^.

In conclusion, MS is associated with excess NfL-age trajectory, producing an older biological profile. This holds implications in clinical practice when making age-related considerations, and in clinical trials to understand, for instance, the reparatory profile^52^. Overall, our study suggests that NfL-derived age reflects the burden of neuro-axonal loss and may help identify patients with disproportionate biological brain ageing, much better and significantly than chronological age. As such, NfL-derived age may serve as a scalable, blood-based proxy of neuroaxonal aging in MS, with potential implications for risk stratification and therapeutic timing in an increasingly aging population.

## Supplementary Information

Below is the link to the electronic supplementary material.

Supplementary file1 (DOCX 14 KB)

Supplementary file2 (DOCX 16 KB)

Supplementary file3 (DOCX 16 KB)

Supplementary file4 (DOCX 16 KB)

Supplementary file5 (DOCX 15 KB)

Supplementary file6 (DOCX 20 KB)

## Data Availability

Data are available upon reasonable request to the corresponding author.
