# Supplementary material for: Evaluating the neurofilament light chain-derived gap between biological and chronological age in multiple sclerosis and controls
Source: J Neurol. 2026 Sep 27;273(10):625. doi: 10.1007/s00415-026-14157-6 (PMC13616846; doi:10.1007/s00415-026-14157-6)
Supplement: Supplementary file 1 [file 415_2026_14157_MOESM1_ESM.docx]

**Figure S1. Differences between chronological age and NfL-derived age.**

Sankey diagrams show the redistribution of participants from chronological age groups to plasma NfL‑derived age categories in pwMS without EDSS Progression (A) and controls (B). Chronological age groups and NfL‑derived age categories were defined according to age‑specific pNfL cut‑offs (5–<17, 18–<50, 51–<61, 60–<70, and ≥70 years). The “over” category indicates individuals whose NfL‑derived age category exceeds their corresponding chronological age group based on these cut‑offs. Thus, participants classified as “over” have pNfL levels above the expected threshold for their age band, reflecting a higher NfL‑derived age relative to their chronological age.

Panel C shows the distribution of age gaps across pwMS without EDSS Progression (orange) and controls (blue), displayed through overlaid histograms and kernel density estimates.
