# Supplementary material for: Evaluating the neurofilament light chain-derived gap between biological and chronological age in multiple sclerosis and controls
Source: J Neurol. 2026 Sep 27;273(10):625. doi: 10.1007/s00415-026-14157-6 (PMC13616846; doi:10.1007/s00415-026-14157-6)
Supplement: Supplementary file 2 [file 415_2026_14157_MOESM2_ESM.docx]

**Table S1.** Chronological and NfL-derived age groups in MS cases and controls.

|  | **MS (n = 792)** | **Controls (n = 302)** | **Total (n = 1,094)** |
| --- | --- | --- | --- |
| **Chronological age groups** |  |  |  |
| 18–50, N (%) | 472 (59.6%) | 154 (51.0%) | 626 (57.2%) |
| 51–60, N (%) | 230 (29.0%) | 48 (15.9%) | 278 (25.4%) |
| 61–70, N (%) | 86 (10.9%) | 45 (14.9%) | 131 (12.0%) |
| ≥70, N (%) | 4 (0.5%) | 55 (18.2%) | 59 (5.4%) |
| **NfL-derived age groups** |  |  |  |
| 5–17, N (%) | 167 (21.1%) | 91 (30.1%) | 258 (23.6%) |
| 18–50, N (%) | 258 (32.6%) | 78 (25.8%) | 336 (30.7%) |
| 51–60, N (%) | 246 (31.1%) | 53 (17.6%) | 299 (27.3%) |
| 61–70, N (%) | 68 (8.6%) | 33 (10.9%) | 101 (9.2%) |
| 70+, N (%) | 44 (5.6%) | 28 (9.3%) | 72 (6.6%) |
| Over, N (%) | 9 (1.1%) | 19 (6.3%) | 28 (2.6%) |
| **NfL age gap** |  |  |  |
| −3, N (%) | 2 (0.3%) | 2 (0.7%) | 4 (0.4%) |
| −2, N (%) | 42 (5.3%) | 17 (5.6%) | 59 (5.4%) |
| −1, N (%) | 256 (32.3%) | 132 (43.7%) | 388 (35.5%) |
| 0, N (%) | 266 (33.6%) | 103 (34.1%) | 369 (33.7%) |
| +1, N (%) | 155 (19.6%) | 42 (13.9%) | 197 (18.0%) |
| +2, N (%) | 56 (7.1%) | 4 (1.3%) | 60 (5.5%) |
| +3, N (%) | 12 (1.5%) | 1 (0.3%) | 13 (1.2%) |
| +4, N (%) | 3 (0.4%) | 1 (0.3%) | 4 (0.4%) |
