# Supplementary material for: Evaluating the neurofilament light chain-derived gap between biological and chronological age in multiple sclerosis and controls
Source: J Neurol. 2026 Sep 27;273(10):625. doi: 10.1007/s00415-026-14157-6 (PMC13616846; doi:10.1007/s00415-026-14157-6)
Supplement: Supplementary file 3 [file 415_2026_14157_MOESM3_ESM.docx]

**Table S2. Demographic, laboratory, and clinical features of MS cases without EDSS progression and controls**

|  | **MS (n = 692)** | **Controls (n = 302)** | **p value** | **Total (n = 994)** |
| --- | --- | --- | --- | --- |
| **Age, years (mean ± SD)** | 46.72 ± 11.07 | 50.61 ± 18.11 | <0.001 | 47.90 ± 13.71 |
| **Sex, female (n, %)** | 445 (64.3%) | 157 (52.0%) | <0.001 | 602 (60.6%) |
| **Disease duration, years (mean ± SD)** | 16.25 ± 9.59 | — | — | — |
| **Follow-up, years (mean ± SD)** | 1.97 ± 0.49 | — | — | — |
| **EDSS, median (range)** | 3 (1–8) | — | — | — |
| **Disease course** |  |  |  |  |
| Relapsing (n, %) | 543 (78.5%) | — | — | — |
| Progressive (n, %) | 149 (21.5%) | — | — | — |
| **DMT group** |  |  |  |  |
| Injectable (n, %) | 60 (8.7%) | — | — | — |
| Oral (n, %) | 431 (62.2%) | — | — | — |
| Monoclonal antibodies (n, %) | 201 (29.1%) | — | — | — |
| **pNfL (pg/ml, mean ± SD)** | 11.78 ± 8.48 | 14.71 ± 15.33 | <0.001 | 12.67 ± 11.10 |
| **Simoa-converted NfL (pg/mL, mean ± SD)** | 11.02 ± 7.09 | 13.46 ± 12.83 | <0.001 | 11.76 ± 9.28 |
