# Supplementary material for: Evaluating the neurofilament light chain-derived gap between biological and chronological age in multiple sclerosis and controls
Source: J Neurol. 2026 Sep 27;273(10):625. doi: 10.1007/s00415-026-14157-6 (PMC13616846; doi:10.1007/s00415-026-14157-6)
Supplement: Supplementary file 4 [file 415_2026_14157_MOESM4_ESM.docx]

**Table S3.** Distribution of participants across chronological age groups, NfL‑derived age categories, and age‑gap classes in pwMS without EDSS progression(n=692) and controls (n=302), with overall totals for the full cohort (n=994).

|  | **MS (n = 692)** | **Controls (n = 302)** | **Total (n = 994)** |
| --- | --- | --- | --- |
| **Chronological age groups** |  |  |  |
| 18–50, N (%) | 432 (62.4%) | 154 (51.0%) | 586 (59.0%) |
| 51–60, N (%) | 192 (27.8%) | 48 (15.9%) | 240 (24.1%) |
| 61–70, N (%) | 66 (9.5%) | 5 (14.9%) | 111 (11.2%) |
| ≥70, N (%) | 2 (0.3%) | 55 (18.2%) | 57 (5.7%) |
| **NfL-derived age groups** |  |  |  |
| 5–17, N (%) | 167 (21.1%) | 91 (30.1%) | 258 (23.6%) |
| 18–50, N (%) | 234 (33.8%) | 78 (25.8%) | 312 (31.4%) |
| 51–60, N (%) | 206 (29.8%) | 53 (17.5%) | 259 (26.1%) |
| 61–70, N (%) | 57 (8.2%) | 33 (10.9%) | 90 (9.1%) |
| 70+, N (%) | 35 (5.1%) | 28 (9.3%) | 63 (6.3%) |
| Over, N (%) | 8 (1.2%) | 19 (6.3%) | 27 (2.7%) |
| **NfL age gap** |  |  |  |
| -3, N (%) | 1 (0.1%) | 2 (0.7%) | 3 (0.3%) |
| -2, N (%) | 36 (5.2%) | 17 (5.6%) | 53 (5.3%) |
| -1, N (%) | 220 (31.8%) | 132 (43.7%) | 352 (35.4%) |
| 0, N (%) | 244 (35.3%) | 103 (34.1%) | 347 (34.9%) |
| 1, N (%) | 130 (18.8%) | 42 (13.9%) | 172 (17.3%) |
| 2, N (%) | 46 (6.7%) | 4 (1.3%) | 50 (5.0%) |
| 3, N (%) | 12 (1.7%) | 1 (0.3%) | 13 (1.3%) |
| 4, N (%) | 3 (0.4%) | 1 (0.3%) | 4 (0.4%) |
