# Supplementary material for: Evaluating the neurofilament light chain-derived gap between biological and chronological age in multiple sclerosis and controls
Source: J Neurol. 2026 Sep 27;273(10):625. doi: 10.1007/s00415-026-14157-6 (PMC13616846; doi:10.1007/s00415-026-14157-6)
Supplement: Supplementary file 5 [file 415_2026_14157_MOESM5_ESM.docx]

|  | **OR** | **95% CI Lower** | **95% CI Upper** | **p value** |
| --- | --- | --- | --- | --- |
| Controls | 7.23 | 5.40 | 9.69 | **<0.001** |
| pwMS (5-17 years) | 4.75 | 2.98 | 7.56 | **<0.001** |
| pwMS (18-50 years) | 2.54 | 1.99 | 3.23 | **<0.001** |
| pwMS (51-60 years) | 2.21 | 1.66 | 2.95 | **<0.001** |
| pwMS (61-70 years) | 2.52 | 1.69 | 3.75 | **<0.001** |
| pwMS (≥70 years) | 3.21 | 1.25 | 8.20 | **0.015** |
| pwMS vs Controls | 1.44 | 1.10 | 1.90 | **0.008** |

**Table S3.** **Association between chronological age group and NfL-derived age group in controls and pwMS.** The table reports odds ratios (ORs), 95% confidence intervals (95% CIs), and p values forthe association between chronological age group and NfL-derived age group. For pwMS, ORs represent threshold-specific cumulative odds ratios derived from generalized ordered logistic models. Significant results (p < 0.05) are reported in bold.
