# Supplementary material for: Evaluating the neurofilament light chain-derived gap between biological and chronological age in multiple sclerosis and controls
Source: J Neurol. 2026 Sep 27;273(10):625. doi: 10.1007/s00415-026-14157-6 (PMC13616846; doi:10.1007/s00415-026-14157-6)
Supplement: Supplementary file 6 [file 415_2026_14157_MOESM6_ESM.docx]

**Table S5.** Table shows coefficients (Coeff), odds ratio (OR), 95% confidence intervals (95% CI), and p values for the associations between clinical predictors and chronological age group, NfL‑derived age group and brain age gap as dependent variables. and clinical variable (disease duration, EDSS, descriptor of disease progression, DMT group, DMT duration, number of previous DMTs) in turn as independent variables in pwMS without EDSS progression. Covariates for regression models were age and sex. For disease duration, ORs represent threshold-specific cumulative odds ratios derived from generalized ordered logistic models. Significant results (p < 0.05) are reported in bold.

|  |  | **OR / Coeff** | **95% CI Lower** | **95% CI Upper** | **p value** |
| --- | --- | --- | --- | --- | --- |
| **EDSS** | Chronological age group | **1.55** | 1.41 | 1.70 | **<0.001** |
|  | NfL-derived age | **1.49** | 1.37 | 1.63 | **<0.001** |
|  | NfL age gap (adj. age, sex) | **0.15** | 0.10 | 0.21 | **<0.001** |
|  |  |  |  |  |  |
| **Disease Duration (18–50 yrs)** | Chronological age group | 1.12 | 1.10 | 1.14 | **<0.001** |
|  | NfL-derived age | 1.05 | 1.03 | 1.06 | **<0.001** |
|  | NfL age gap | 0.01 | –0.00 | 0.02 | 0.164 |
| **Disease Duration (51–60 yrs)** | Chronological age group | 1.08 | 1.05 | 1.11 | **<0.001** |
|  | NfL-derived age | 1.05 | 1.03 | 1.06 | **<0.001** |
|  | NfL age gap | 0.01 | –0.00 | 0.02 | 0.164 |
| **Disease Duration (61–70 yrs)** | Chronological age group | **1.24** | 1.12 | 1.38 | **<0.001** |
|  | NfL-derived age | 1.05 | 1.03 | 1.06 | **<0.001** |
|  | NfL age gap | 0.01 | –0.00 | 0.02 | 0.164 |
| **Disease Duration (>70 yrs)** | Chronological age group | — | — | — | — |
|  | NfL-derived age | 1.05 | 1.03 | 1.06 | **<0.001** |
|  | NfL age gap | 0.01 | –0.00 | 0.02 | 0.164 |
|  |  |  |  |  |  |
| **Clinical course (Progressive vs Relapsing)** | hronological age group | **2.33** | 1.64 | 3.30 | **<0.001** |
|  | NfL-derived age | **2.47** | 1.78 | 3.45 | **<0.001** |
|  | NfL age gap | **0.40** | 0.19 | 0.60 | **<0.001** |
|  |  |  |  |  |  |
| **DMT group (ref: Injectable)** |  |  |  |  |  |
| **Injectable (reference)** |  |  |  |  |  |
| **Oral** | Chronological age group | **0.37** | 0.22 | 0.62 | **<0.001** |
|  | NfL-derived age | **0.52** | 0.32 | 0.83 | **0.006** |
|  | NfL age gap | –0.031 | –0.332 | 0.271 | 0.842 |
| **Monoclonal** | Chronological age group | **0.41** | 0.24 | 0.72 | **0.002** |
|  | NfL-derived age | 0.78 | 0.47 | 1.29 | 0.336 |
|  | NfL age gap | 0.227 | –0.093 | 0.547 | 0.163 |
|  |  |  |  |  |  |
| **DMT duration** | Chronological age group | **1.10** | 1.06 | 1.14 | **<0.001** |
|  | NfL-derived age | **1.06** | 1.02 | 1.09 | **0.001** |
|  | NfL age gap | 0.004 | –0.017 | 0.025 | 0.738 |
|  |  |  |  |  |  |
| **Number of previous DMTs** | Chronological age group | 1.01 | 0.91 | 1.11 | 0.917 |
|  | NfL-derived age | 1.05 | 0.96 | 1.15 | 0.300 |
|  | 0.055 | –0.001 | 0.111 | 0.056 | 0.055 |
